# Iron Mobilization from Ferritin in Yeast Cell Lysate and Physiological Implications

**DOI:** 10.3390/ijms23116100

**Published:** 2022-05-29

**Authors:** Gideon L. Smith, Ayush K. Srivastava, Aliaksandra A. Reutovich, Nathan J. Hunter, Paolo Arosio, Artem Melman, Fadi Bou-Abdallah

**Affiliations:** 1 Department of Chemistry, State University of New York, Potsdam, NY 13676, USA; smithgl201@potsdam.edu (G.L.S.); srivasak@potsdam.edu (A.K.S.); reutovaa202@potsdam.edu (A.A.R.); hunternj@potsdam.edu (N.J.H.); 2 Department of Molecular & Translational Medicine, University of Brescia, 25121 Brescia, Italy; paolo.arosio@unibs.it; 3 Department of Chemistry & Biomolecular Science, Clarkson University, Potsdam, NY 13699, USA; amelman@clarkson.edu

**Keywords:** ferritin, yeast cell lysate, iron mobilization, kinetics, flavoenzymes, NADPH, FMN, FAD, riboflavin, labile iron pool

## Abstract

Most in vitro iron mobilization studies from ferritin have been performed in aqueous buffered solutions using a variety of reducing substances. The kinetics of iron mobilization from ferritin in a medium that resembles the complex milieu of cells could dramatically differ from those in aqueous solutions, and to our knowledge, no such studies have been performed. Here, we have studied the kinetics of iron release from ferritin in fresh yeast cell lysates and examined the effect of cellular metabolites on this process. Our results show that iron release from ferritin in buffer is extremely slow compared to cell lysate under identical experimental conditions, suggesting that certain cellular metabolites present in yeast cell lysate facilitate the reductive release of ferric iron from the ferritin core. Using filtration membranes with different molecular weight cut-offs (3, 10, 30, 50, and 100 kDa), we demonstrate that a cellular component >50 kDa is implicated in the reductive release of iron. When the cell lysate was washed three times with buffer, or when NADPH was omitted from the solution, a dramatic decrease in iron mobilization rates was observed. The addition of physiological concentrations of free flavins, such as FMN, FAD, and riboflavin showed about a two-fold increase in the amount of released iron. Notably, all iron release kinetics occurred while the solution oxygen level was still high. Altogether, our results indicate that in addition to ferritin proteolysis, there exists an auxiliary iron reductive mechanism that involves long-range electron transfer reactions facilitated by the ferritin shell. The physiological implications of such iron reductive mechanisms are discussed.

## 1. Introduction

Iron is a critical nutrient required for most forms of life and participates in many biological processes. However, too much or too little iron can be a problematic and significant contributor to human diseases. Iron not immediately utilized by cells is stored in ferritin, a ubiquitous 24-mer iron biomineralizing protein that plays a critical role in iron homeostasis and serves as the principal reservoir of metabolic iron within the cell [1,2,3,4]. While considerable progress has been achieved towards understanding the ferritin in-vitro iron sequestration mechanisms [3,4,5,6,7,8,9,10,11,12], iron mobilization is not sufficiently understood and is rather controversial [13,14,15,16,17,18,19,20]. In vitro (or in cell-free systems), the rates of iron uptake and oxidation depend on the concentration of ferritin, its iron content, and the concentration of added Fe(II) cations, but not on the concentration of oxygen [8]. However, it is still unclear how iron is delivered to ferritin in vivo and how it is mobilized. Recent mammalian cells studies have shown that delivery of cytosolic Fe(II) ions to specific iron proteins, including ferritin, is facilitated by the poly rC-binding protein (PCBP) family of proteins [21,22,23,24,25], particularly PCBP1 (and to a lesser extent PCBP2), both of which are abundantly expressed in all human tissues. On the other hand, lysosomal ferritin proteolysis has been shown to be the primary mechanism for iron mobilization from ferritin, where the degradation of the ferritin shell occurs through a specific mechanism (called ferritinophagy) that involves a nuclear receptor coactivator-4 (NCOA4) [26,27,28,29]. However, it is still unknown whether the ferritin autophagic degradation process is the only mechanism by which iron is released from the protein or whether other auxiliary iron mobilization mechanisms that provide an immediate and more economical source of iron to cells exist. Given the reducing environment of the cell, it is conceivable that under physiological concentrations of oxygen, such auxiliary iron reductive mechanisms may be important pathways to maintain a constant concentration of labile iron (i.e., <1 µM) so that the iron uptake and release processes could balance each other out for optimal cellular functions. Nonetheless, the current understanding of the mechanisms of iron reductive mobilization from ferritin and their physiological implications have been discussed in several excellent literature reviews [13,30,31,32].

Despite potential advantages of this reductive iron mobilization pathway from intact ferritin, and the suggestion that it can play a role in response to oxidative stress [33,34,35,36,37,38,39,40], there has been no proof that the reaction is physiologically relevant. We think one important factor to consider when studying in vivo iron mobilization mechanisms is the ability of an endogenous reducing agent to reduce ferritin iron under normoxic (and quantifiable) concentrations of oxygen, and in the presence of cellular metabolites and oxidoreductase enzymes which are ubiquitous in every living cell. Attempts to conduct these experiments using a living cell model are challenging and present significant experimental hurdles. Not only is the concentration of ferritin in living cells miniscule, but the intracellular oxygen concentration is hard to follow or measure with high precision. Furthermore, the measurement and speciation of very small concentrations of intracellular iron cations are difficult to track and can be skewed by the addition of exogenous iron chelators. To circumvent the experimental challenges of working with living cells and examine the relevance of iron reductive mobilization from ferritin under physiologically relevant conditions, a simple system consisting of freshly prepared homogeneous yeast cell lysate solutions is chosen, based on documented similarities of iron metabolism processes between animals and fungi and the presence of cellular metabolites that have been excluded in prior in vitro iron mobilization studies.

Our hypothesis is that in addition to the generally accepted iron mobilization mechanisms through ferritin proteolytic degradation, there exists an auxiliary iron reductive mechanism that utilizes long-range electron transfer pathways, facilitated by the ferritin shell. Furthermore, the iron mobilization rates in cell lysate can substantially differ from those in aqueous buffered solutions due to the presence of yet unknown cellular metabolites. Although our study does not make any assumptions or predictions about the nature of iron-reducing species or oxidoreductase enzymes and their cofactors, our objective is to investigate these possibilities using yeast cell lysate under normoxic conditions and explore whether such an iron mobilization mechanism exists in cells.

## 2. Results

### 2.1. Iron Mobilization from Ferritin in Cell Lysate

**Figure 1:**
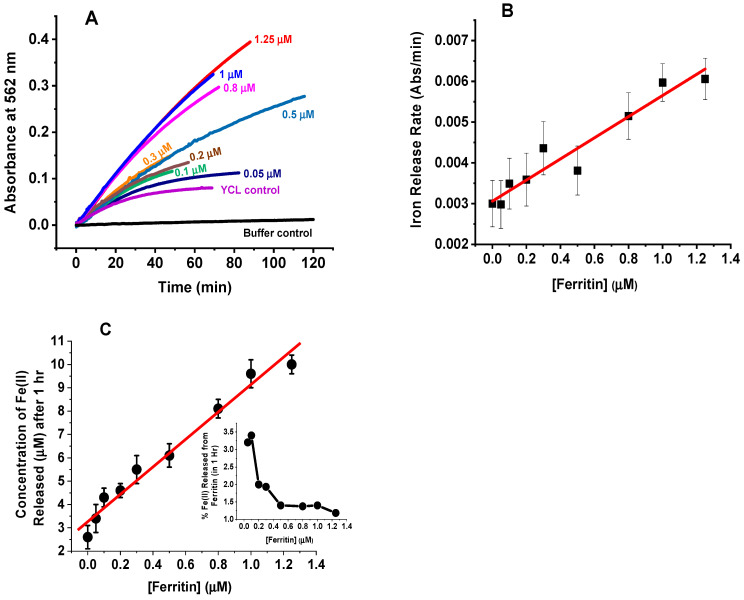
Iron mobilization kinetics from ferritin in yeast cell lysate (YCL). (**A**) Absorbance change of the (Fe^2+^–(ferrozine)_3_) complex following the exogenous addition of ferritin and NADPH to yeast cell lysate. (**B**) Rates of iron release as a function of ferritin concentration. (**C**) Concentration of released iron, in one hour, as a function of ferritin, with % Fe(II) released shown in the inset. Conditions: 555 µL YCL, 1.5 mM NADPH, reaction volume 1 or 2.3 mL, ferritin concentration is indicated next to each kinetic trace, 3 mM ferrozine, 20 mM MOPS, pH 7.4. The red lines are first-order fits to the data, and the ferritin sample used here is recombinant human heteropolymer ferritin H_23_L_1_ loaded with 500 Fe/shell.

To examine whether iron is reductively removed from ferritin in yeast cell lysate (YCL), iron release kinetics were followed by light absorption spectroscopy at 562 nm, where the (Fe^2+^–(ferrozine)_3_) complex absorbs [41]. Figure 1A shows an increase in absorbance over time, suggesting that ferrous ions are slowly released in the medium and form an (Fe^2+^–(ferrozine)_3_) complex. The rates of iron mobilization (Figure 1B) and the concentration of released iron monitored over one hour (Figure 1C) were found to increase linearly with increased ferritin concentration, suggesting that the observed kinetics are those of iron reduction and release from ferritin. The percent of iron release from ferritin varied between 1 and 3% and reached a plateau at ferritin concentrations > 0.5 µM (Figure 1C, inset). Under the same experimental conditions but in the absence of cell lysate, no absorbance change was observed (data not shown), indicating that the reductive release of iron from ferritin is due to the presence of reducing agents and cellular metabolites in YCL.

### 2.2. Iron Release Kinetics and the Effect of Cellular Metabolites

**Figure 2:**
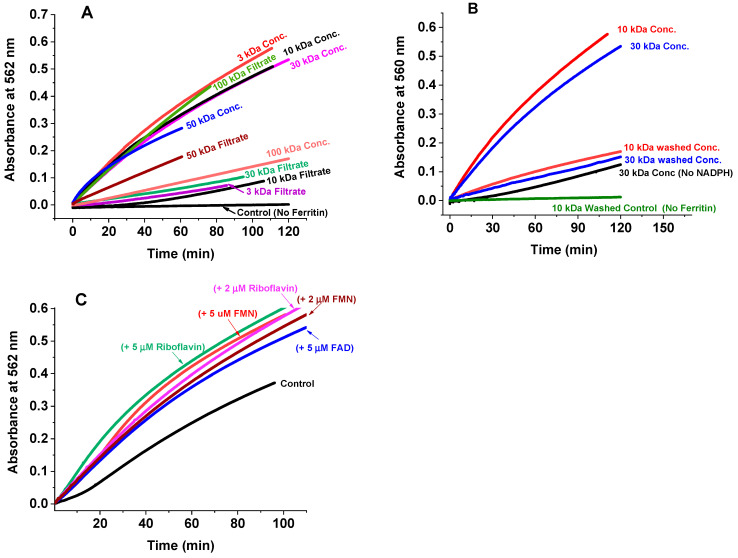
Iron reduction and release kinetics monitored by UV-Vis using cell lysate filtrate or retentate solutions separated using different MW membrane cut-offs (3, 10, 30, 50, or 100 kDa). (**A**) The control-subtracted iron release kinetics of the partitioned cell lysate solutions.(**B**) In the absence of exogenously added NADPH or ferritin, the iron release kinetics. (**C**) The kinetics of iron release were followed in the presence of 2–5 µM FMN, FAD, or riboflavin using freshly prepared whole cell lysate solutions. Conditions: 555 µL of the retentates or filtrates, 1.5 mM NADPH, 0.8 µM homopolymer H-ferritin or heteropolymer ferritins (H19:L5, H22:L2) loaded with 500Fe(III)/shell, 3 mM ferrozine, 2–5 µM FMN, FAD or riboflavin, 20 mM MOPS, pH 7.4. The displayed kinetics are averages of at least 2 different trials. The control kinetic experiment using cell lysate in the absence of ferritin is similar to that of buffer with ferritin.

To reveal the molecular size of the cellular metabolites responsible for ferritin iron reduction, freshly prepared cell lysate solutions were separated into retentate and filtrate using a pressurized Amicon concentrator fitted with different Millipore filtration membranes of varying pore sizes (i.e., 3, 10, 30, 50, or 100 kDa MW cut-off). The control-subtracted iron release kinetics of the partitioned cell lysate solutions are displayed in Figure 2A. The filtrates of 3, 10, and 30 kDa (and the retentate of 100 kDa) membranes exhibited an absorbance five-fold lower than that of the corresponding retentates (and filtrate) solutions, respectively, suggesting that the species responsible for the iron-induced release from ferritin has an MW greater than 30 kDa, but less than 100 kDa. The retentate of the 50 kDa membrane cut-off was only about 1.5-fold higher than the corresponding filtrate, indicating that the molecular species has an MW in the 50 kDa range. Interestingly, in the absence of exogenously added NADPH or ferritin, the iron release kinetics were similar to the filtrates of 3, 10, and 30 kDa MW cut-off (Figure 2B).

In a different series of experiments, the retentates of 10 and 30 kDa (3 mL) were washed four times with 20 mM MOPS buffer pH 7.4 in the pressurized Amicon concentrator (each time adding an equal volume of buffer and concentrating it down to 3 mL) and then used to test their efficacy in releasing iron from ferritin (Figure 2B). The washing caused a major reduction of the iron release kinetics, which were similar to those of the retentates without added NADPH, suggesting that low molecular weight metabolites, enzyme cofactors, or free flavins are required for efficient iron release. To further test this hypothesis, the kinetics of iron release were followed in the presence of 2–5 µM FMN, FAD, or riboflavin using freshly prepared whole cell lysate solutions. As seen in Figure 2C, an approximately two-fold increase in absorbance is observed with the three types of flavins, irrespective of the type of ferritins used (i.e., homopolymer H-ferritin or heteropolymer ferritin with different H to L ratios, i.e., H19:L5, H22:L2), suggesting that exogenously added free flavins with a concentration close to physiological can enhance electron transfers from NAD(P)H to ferric iron in the iron storage proteins for a more effective release of ferrous ions.

### 2.3. Effect of Temperature on the Iron Release Kinetics and Differential Scanning Calorimetry (DSC) of Cell Lysate

**Figure 3:**
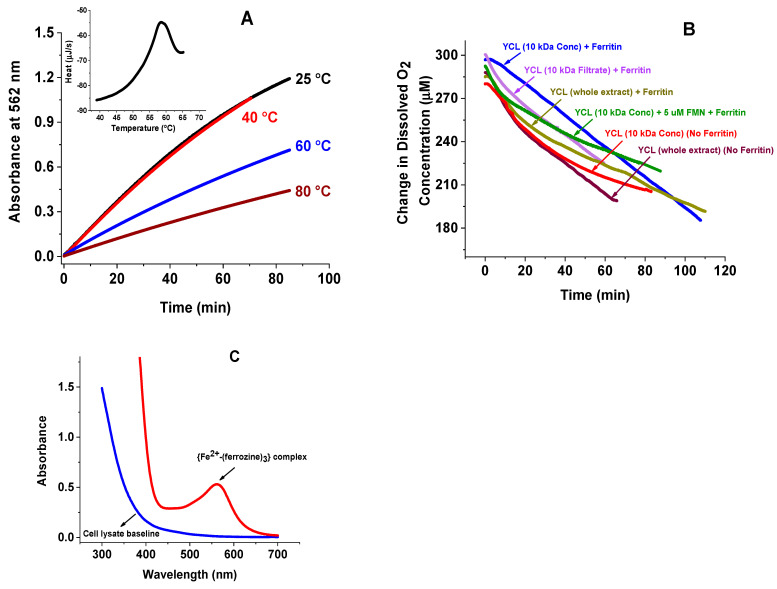
(**A**) Iron release kinetics as a function of temperature. ((**A**), Inset) Differential scanning calorimetry of whole cell lysate. (**B**) Representative oxygen consumption kinetics of different YCL solutions (whole cell lysate, retentate, and filtrate from a 10 kDa membrane cut-off); control experiments of whole YCL with and without added ferritin are also included. (**C**) Example UV–vis absorption spectrum of a solution at the end of a kinetic run. All other experimental conditions are the same as those in Figure 1 and Figure 2.

To prove that the cellular species that facilitates iron release from ferritin is a protein/enzyme, and that the observed activity is thermosensitive, freshly prepared cell lysate samples were heated at 40, 60, and 80 °C for 30 min, and the iron release kinetics performed. Compared to the control, the 60 and 80 °C heated samples showed a significant reduction (40 and 64%, respectively) in iron release (Figure 3A). Differential scanning calorimetry (DSC) of the whole cell lysate showed an average melting temperature of (57.7 ± 3.2) °C, consistent with the loss of activity observed with the kinetic data (Figure 3A, inset).

### 2.4. Oxygen Concentration and O_2_ Consumption Kinetics

Earlier in vitro iron mobilization kinetics employing the non-enzymatic NADH and FMN system showed that iron release from ferritin occurs only after most of the dissolved oxygen has been depleted from the solution [15,16,32]. The rapid re-oxidation of the reduced agent (i.e., FMNH_2_ in this case) by O_2_, even at a low concentration of dissolved oxygen prevented any appreciable release of iron from ferritin. To explore whether O_2_ plays a role during iron mobilization from ferritin in cell lysate, a micro-oxygen electrode was used to monitor the level of oxygen during the iron release experiments. Figure 3B shows a series of oxygen consumption kinetics under the same experimental conditions as Figure 1 and Figure 2. Regardless of the concentration or type of proteins used (homopolymer or heteropolymer ferritin), cell lysate retentate or filtrate, with or without FMN or FAD, the iron mobilization kinetics occurred in the presence of a significant concentration of dissolved O_2_. The O_2_ level slowly decreased over the course of the reaction to reach values no less than 180 µM, two hours later. These results suggest that in vivo iron mobilization mechanisms could still occur under normoxic conditions, and more likely so under hypoxia, as shown in our buffer experiments [15,16,32], where concentrations of less than 10 µM O_2_ promoted a rapid iron release from ferritin. These O_2_ concentrations are well below the typical physiological concentration of ~5% oxygen or ~50 µM [42] in normoxic cells. Lastly, UV-vis spectra of solutions collected at the end of the kinetics showed the characteristic pink color and typical 560 nm maximum absorbance of (Fe^2+^–(ferrozine)_3_) complexes (Figure 3C) [41].

## 3. Discussion

The release of ferritin iron is crucial for various cellular metabolic activities. Numerous in vitro studies and different mechanisms of iron release from ferritin have been recently reviewed [13,19,26,30,31,32,43]. In most cases, large concentrations of reducing agents and/or suitable electron transfer mediators have been used, with little to no physiological relevance. Nonetheless, there are three different models of iron release that have been proposed and are represented by (1) lysosomal ferritin degradation, (2) direct iron removal, and (3) reductive-chelation mechanisms. While the first model is the most accepted to date, little evidence in support of the second and third models exist. However, considering the reducing nature of the intracellular environment and many in vitro studies showing highly efficient iron mobilization mechanisms via the reductive method, more insights have started to emerge in favor of the reduction model [13,43]. Furthermore, a careful balance between the rates of iron mineralization and those of iron mobilization in vivo requires a delicate balance between these two processes to maintain a low micromolar concentration of labile iron [32]. Paradoxically, ferritin proteolytic degradation and biosynthesis appear to occur on a different time scale compared to iron mineralization, raising the possibility that a continuous and slow non-regulated process of iron mobilization (perhaps through iron reduction or chelation) might be at play to offset the slow process of ferritin expression and proteolysis.

In addition to low molecular weight physiological reducing agents such as thiols, ascorbate, and glutathione that are capable of reducing the iron core of ferritin [43], reduced flavins (such as FMNH_2_, FADH_2_, and reduced riboflavins) have been shown to be the most potent ferritin reducing agents in cell-free systems [15,16]. Their presence in the cytoplasm raises the possibility of likely candidates for the reductive iron mobilization from endogenous cytoplasmic ferritins [32,44,45,46,47,48,49], even though the physiological relevance of such mechanism is unclear, particularly that free reduced flavins that are not protein-bound are rapidly oxidized by molecular oxygen [45]. While FMN can be reduced back to FMNH_2_ by cellular NAD(P)H:flavin oxidoreductases [46], the rate constants for the re-oxidation of reduced flavins by molecular oxygen are very high, suggesting that the stationary concentrations of reduced flavins are extremely low. A recent study confirmed the presence of free flavins in the cytosol of *A. xylanus*, a bacterium that grows well under both aerobic and anaerobic conditions, and estimated their intracellular concentrations at 8 µM FAD, 3 µM FMN, and 1 µM riboflavin [50]. Two flavoproteins (an NADH oxidase and NAD(P)H oxidoreductase) reduced the FAD and FMN to FADH_2_ and FMNH_2_, which are used to generate ferrous ions necessary for the aerobic growth of the bacterium. Other bacterial ferric reductases, both flavin-dependent and flavin-independent, oxygen-sensitive and oxygen-insensitive [47,51], have also been shown to reduce and mobilize iron from physiological ferric complexes such as Fe(III)-siderophores and also from the iron storage protein ferritin [51]. Similarly, using rat and turkey liver homogenates [52], it was found that xanthine, NADH, or NADPH are essential reducing substrates for at least two enzyme systems (xanthine oxidoreductase and NAD(P)H oxidoreductase) and that flavin nucleotides such as FMN and FAD were required to shuttle electrons from the reduced enzymes to the ferritin iron for iron reduction and mobilization. Both ferritin iron core formation and reductive iron mobilization processes have been shown to involve electron transfer reactions through the ferritin shell [53,54,55,56]. These observations are consistent with our own results with yeast cell lysate (Figure 2), indicating that specific intracellular iron reducing species such as FMNH_2_, FADH_2_, and/or reduced riboflavin could facilitate the in vivo reduction of the ferritin iron core. However, further work is needed to confirm the identity of the reducing substrates, the specific enzymes (flavoenzymes or oxidoreductase systems) involved, and the molecular mechanism of the iron reductive mobilization in eukaryotes. Additionally, the effect of physiological oxygen concentrations, varying pH levels, and other cellular reducing agents such as ascorbate, glutathione, cysteine, etc. could play important roles in the mechanisms of iron mobilization from ferritin since their cellular concentrations and regulation may be affected by cellular iron status and other cellular conditions.

## 4. Materials and Methods

Ferrous sulfate heptahydrate was purchased from J.T. Baker Chemical (Radnor, PA, USA), MOPS from Sigma-Aldrich (St. Louis, MO, USA) FMN and FAD from Cayman Chemical Company (Ann Arbor, MI, USA), NADPH from EMD Millipore Corporation (Burlington, MA, USA), riboflavin from TCI Chemicals (Tokyo, Japan), and ferrozine from Alfa Aesar (Haverhill, MA, USA).

### 4.1. Ferritin Expression and Purification

Recombinant human homopolymer and heteropolymer ferritins were produced in *E. coli* Rosetta-gami B strain as described in detail elsewhere [57]. Protein purification and quantification were performed according to established protocols using Sepharose 6B and Superdex 200 size exclusion chromatography (Akta Go, Cytiva, GE Healthcare, Marlborough, MA, USA), native and SDS-PAGE, and capillary gel electrophoresis (7100 model from Agilent Technologies, Santa Clara, CA, USA). Protein concentrations were determined using the Micro BCA Protein Assay Kit (Thermo Fisher Scientific, Waltham, MA, USA) and a Varioskan LUX microplate reader from Thermo Fisher Scientific.

### 4.2. Iron Loading into Ferritin

Iron solutions were prepared by dissolving ferrous iron sulfate in deionized water, adjusted to pH 2.0 with hydrochloric acid, and then filtered through 0.2 µm Whatman Puradisc polyethersulfone sterile syringe filters from Cytiva. Typically, 5 µL injections of a stock ferrous sulfate solution are added to a 1 mL protein solution prepared in 20 mM Mops, pH 7.4, sitting in a quartz cuvette with a screw-on cap stock to achieve a desired iron/protein ratio. The Fe(II) oxidation kinetics were followed under constant stirring on a Varian Cary 50 Bio or Cary 60 spectrophotometers from Agilent Technologies at 305 nm, where the Fe(III) oxo(hydroxo) species absorbs.

### 4.3. Yeast Cell Growth

*S. cerevisiae* (W303-1A) cells preserved in glycerol at −80 °C were provided by Dr. F. Nazeer in the Chemistry department at SUNY Potsdam. Cells were inoculated in a 10 mL YPD broth (2% Peptone, 1% Yeast Extract, 2% Dextrose) from Teknova (Hollister, CA, USA) and incubated overnight in an orbital shaker at 30 °C with 150 rpm agitation. Overnight cell culture (10–50 µL) was taken to streak on a YPD-Agar Petri dish, which was then incubated at 30 °C until isolated colonies showed growth (24–48 h). The pre-inoculum was prepared by inoculating a single isolated colony into 100 mL of YPD medium and incubated overnight at 30 °C with continuous agitation at 200 rpm. The 100 mL overnight pre-culture was re-inoculated into 1 L of YPD medium, and the OD was monitored every hour. The cells were harvested after 6 to 8 h during the early exponential phase when the OD_(600 nm)_ reached a value of ~0.8 ± 0.2.

### 4.4. Cell Lysate Preparation

Approximately 3 g of wet pellet from harvested yeast cells were mixed with an equivalent mass of glass beads (425–600 μm, Sigma-Aldrich G8772-100G), and the mixture suspended in 7.5 mL of 20 mM MOPS buffer, pH 7.4. The suspension was vortexed for one minute and allowed to chill on ice for one minute; this process was repeated 3 times. After vortexing, the sample was spun down at 12,000 rpm for 15 min. The supernatant was removed and filtered through a 0.2-micron filter from Cytiva, and the pellet was resuspended in another 7.5 mL of buffer, and the above process was repeated one more time. In the end, a total of 15 mL of cell lysate was obtained and immediately used in the experiments. The protein content of the whole cell lysate was determined by the Micro BCA Protein Assay and found to be approximately (46 ± 5) mg or (3.1 ± 0.3) mg/mL. Excess cell lysate not used within 5–6 h was stored at −80 °C, thawed the next day, and re-used with no observed differences. However, if the cell lysate was kept at −80 °C for more than 3 days, some variability in the results was noted. Therefore, the results of this study were obtained from freshly prepared yeast cell lysate. Finally, to maintain the reductive environment of living cells (i.e., NADPH/NADP^+^ ratio > 100), and to compensate for the interruption of the reduction of NADP^+^ to NADPH during the lysis phase, all experiments were conducted in the presence of excess NADPH (1.5 mM). For experiments requiring fractionation of the cell lysate, approximately 5 mL of freshly prepared lysate was transferred into a pressurized Amicon concentrator (10–15 PSI) using different MW membrane cut-offs from Millipore (3, 10, 30, 50, and 100 kDa) with constant and gentle stirring. Once the original transferred solution was reduced to 1 mL, both the filtrate and the retentate solutions were collected and each diluted with 4 mL of 20 mM MOPS buffer pH 7.4 such that the final volume of each solution was equal to the original volume. The resulting filtrate and retentate solutions were immediately used in the experiments or kept cool on ice until needed.

### 4.5. Iron Mobilization Kinetics in Cell Lysate

Absorption spectroscopy was performed using Varian Cary 50 and Cary 60 UV-vis spectrophotometers from Agilent Technologies. The instruments were zeroed with a reaction mixture containing the cell lysate, a ferritin sample loaded with 500 Fe(III)/shell, ferrozine, and the iron mobilization kinetics were initiated with the addition of NADPH. The reaction was run aerobically but in a capped quartz cuvette to minimize dissolving of atmospheric oxygen while the kinetic ran. They were followed at 562 nm, which is where the (Fe^2+^–(ferrozine)_3_) complex absorbs [42]. Experiments were conducted at 25.0 °C in 20 mM MOPS at pH 7.4. Concentrations of all reagents are given in the Figure captions. Absorbance kinetics were analyzed using Origin Lab 8.0 (OriginLab Corp., Northampton, MA, USA).

### 4.6. Oxygen Microelectrode and O_2_ Kinetics

Dissolved oxygen kinetics were performed in conjunction with the UV-vis kinetics using a 1.0 mL UV-vis quartz cuvette (1 cm path length) with a septum seal cap and a hole drilled through the septum seal to insert the O_2_ probe and minimize the diffusion of atmospheric oxygen inside the solution. The MI-730 micro-oxygen electrode was purchased from Microelectrodes, Inc., (Bedford, NH, USA) and standardized as described elsewhere [3]. The dissolved oxygen data were collected with either an eDAQ isopod (Colorado Springs, CO, USA) or a HoldPeak HP-90EPC voltmeter.

## Data Availability

Not applicable.
